# Allogeneic Hematopoietic Stem Cell Transplantation in Transformed Follicular Lymphoma (tFL): Results of a Retrospective Multicenter Study from GELTAMO/GETH-TC Spanish Groups

**DOI:** 10.3390/cancers14225670

**Published:** 2022-11-18

**Authors:** Beatriz Rey-Búa, Mónica Cabrero, Leyre Bento, Juan Montoro, Mariana Bastos-Oreiro, Rocío Parody, Lucrecia Yañez, Oriana Lopez-Godino, Joud Zanabili, Pilar Herrera, Gonzalo Gutierrez, Ariadna Perez, Jose L. Piñana, Silvana Novelli, María Cortés, Ana Maria Sureda, Dolores Caballero, Alejandro Martín García-Sancho

**Affiliations:** 1 Department of Hematology, Hospital Universitario de Salamanca, IBSAL, CIBERONC, 37007 Salamanca, Spain; 2 Department of Hematology, Hospital Universitario Son Espases, IdISBa, 07120 Palma, Spain; 3 Department of Hematology, Hospital Universitari i Politècnic La Fe, 46026 Valencia, Spain; 4 Department of Hematology, Hospital General Universitario Gregorio Marañón, 28007 Madrid, Spain; 5 Department of Hematology, Institut Català d’Oncologia (ICO), 08908 L’Hospitalet de Llobregat, Spain; 6 Department of Hematology, Hospital Universitario Marqués de Valdecilla, IDIVAL, 39008 Santander, Spain; 7 Department of Hematology, Hospital General Universitario Morales Meseguer, 30008 Murcia, Spain; 8 Department of Hematology, Hospital Universitario Central de Asturias, 33011 Oviedo, Spain; 9 Department of Hematology, Hospital Universitario Ramón y Cajal, 28034 Madrid, Spain; 10 Department of Hematology, Hospital Clínic Barcelona, 08036 Barcelona, Spain; 11 Department of Hematology, Hospital Clínico Universitario de Valencia, 46010 Valencia, Spain; 12 Department of Hematology, Hospital de la Santa Creu i Sant Pau, 08041 Barcelona, Spain; 13 Department of Statistics, Hospital Universitario de Salamanca/IBSAL, 37007 Salamanca, Spain

**Keywords:** non-Hodgkin lymphoma, follicular lymphoma, transformed lymphoma, allogeneic stem cell transplantation

## Abstract

**Simple Summary:**

Follicular lymphoma (FL) is the most prevalent subtype of indolent lymphoma, accounting for 70% of all cases. The estimated risk of histological transformation (tFL), such as diffuse large B cell lymphoma (DLBCL), varies from 2–3% per year to 7–8% at 10 years in different series. Treatment after transformation is not clearly established. Allogeneic hematopoietic stem cell transplantation (alloSCT) could be an option for these patients, but it has not been widely explored. We analyze the efficacy and toxicity of alloSCT in 43 patients from 14 Spanish centers. We observed long-term survival in around one third of the patients, especially those who developed chronic graft versus host disease, indicating that alloSCT continues to be a potentially curative option for patients with tFL, mainly due to the graft versus lymphoma effect.

**Abstract:**

Background: Transformation of follicular lymphoma into an aggressive lymphoma (tFL) worsens the prognosis and the standard treatment is not completely defined. Allogeneic hematopoietic stem cell transplantation (alloSCT) could be a potentially curative option for these patients, but it has not been widely explored. Methods: We designed a retrospective multicenter study to analyze the efficacy and toxicity of alloSCT in tFL patients and potential prognostic factors of survival. Results: A total of 43 patients diagnosed with tFL who underwent alloSCT in 14 Spanish centers between January 2000 and January 2019 were included. Median age was 44 (31–67) years. After a median follow-up of 58 months, estimated 5-year overall survival (OS) and progression-free survival (PFS) were both 35%. Estimated 100-day and 1-year non-relapse mortality (NRM) were 20% and 34%, respectively. The type of conditioning regimen (3-year OS of 52% vs. 20%, respectively, for reduced-intensity vs. myeloablative conditioning) and development of chronic graft versus host disease (cGVHD) (3-year OS of 75% vs. 40%) were the only factors significantly associated with OS. The only variable with an independent association with OS was cGVHD (HR, 3.4; 95% CI, 1.2–9.6). Conclusions: Our results indicate that alloSCT continues to be a potentially curative option for patients with tFL.

## 1. Introduction

Non-Hodgkin’s lymphomas (NHLs) are a heterogeneous group of malignant diseases, ranging from indolent to aggressive lymphomas, with different therapy approaches and prognosis. Follicular lymphoma (FL) is the most prevalent subtype in the former group, accounting for 70% of all cases [1]. It is considered an incurable disease, with a natural history of responses to therapy followed by multiple relapses. Overall survival (OS) of FL patients has improved since the introduction of immunochemotherapy as a first-line treatment [2,3,4]. In fact, those patients who maintain a complete response 30 months (CR30) after induction therapy may have the same prospect of survival as the general population [5]. However, relapse or transformation to a high-grade lymphoma is still the most common cause of death in FL patients, especially after disease transformation [6]. The estimated risk of histological transformation (HT) of FL into an aggressive lymphoma (tFL), such as diffuse large B cell lymphoma (DLBCL), varies from 2–3% per year to 7–8% at 10 years in different series before and after the introduction of rituximab into first-line treatment, and does not have a clear plateau [7,8]. It has customarily been considered a therapy-refractory disease with an aggressive clinical course and poor prognosis, and, although immunochemotherapy has led to better outcomes than in the pre-rituximab era, histological transformation marks a change in FL natural history and significantly worsens patient prognosis [7,8,9,10,11,12].

Treatment after transformation is not clearly established and depends on previous lines of therapy and patient characteristics. In chemosensitive transformation, immunochemotherapy followed by consolidation with high-dose therapy and autologous stem cell transplantation (autoSCT) can lead to long-term remissions, with some series reporting 5-year overall survival (OS) and progression-free survival (PFS) of as much as 50–60% [13,14,15]. However, patients with chemo-refractory transformation or post-autoSCT relapse have limited treatment options [16,17]. New drugs could offer possibilities for these patients, but the exclusion of tFL in most clinical trials suggests that the effect of new, targeted therapies is uncertain in this setting. Chimeric antigen receptor (CAR) T-cell therapy has recently been approved for patients with relapsed or refractory tFL after at least two lines of treatment. This strategy can lead to long-term remissions in 30–40% of patients, although specific data about the efficacy in tFL remain limited, since the cases represent only 16–22% of patients included in pivotal trials [18,19,20,21]. In addition, there are few treatment options for post-CART relapses or non-responding patients.

In this context, despite the availability of new therapies, allogeneic hematopoietic stem cell transplantation (alloSCT) could be the only curative therapy for a group of tFL patients. However, results have not been widely explored as a separate entity in the largest series of alloSCT in lymphoma [22,23,24]. We present the results of a retrospective multicenter study analyzing the role of alloSCT in tFL in what we believe to be the largest series published so far.

## 2. Materials and Methods

### 2.1. Study Design and Objectives

We designed a multicenter retrospective cohort study of patients with a histological diagnosis of DLBCL transformed from FL, who received an alloSCT between January 2000 and January 2019 in twelve Spanish centers that are members of Spanish Groups GELTAMO (Grupo Español de Linfomas y Trasplante Autólogo de Médula Ósea) and GETH-TC (Grupo Español de Trasplante Hematopoyético y Terapia Celular). Informed consent for alloSCT and data collection was obtained locally according to the regulations applicable at the time of transplantation.

Our main objectives were to analyze efficacy (response rates, PFS and OS) and toxicity (engraftment, graft versus host disease [GVHD] and non-relapse mortality [NRM]) of alloSCT in patients with tFL, and to identify potential prognostic factors influencing these outcomes.

### 2.2. Patient Enrollment

Eligible patients were to be identified by the participating centers from local databases. Each center reviewed all the records of patients diagnosed in the period designated and registered all the eligible patients. Data were retrospectively collected from the medical records of the patients. Forty-three patients diagnosed with transformed lymphoma who received an alloSCT were initially collected; four patients were excluded because of a diagnosis other than FL (LLC, *n* = 1; transformed marginal lymphoma, *n* = 3; secondary myelodysplastic syndrome, *n* = 1). Therefore, finally, 38 patients with a diagnosis of follicular lymphoma transformed to DLBCL who received an alloSCT during this period were included in the study.

### 2.3. Study Endpoints and Statistical Analysis

The response to transplantation recorded in the study was that indicated in the medical records based on the clinical judgment of the treating physician, provided it was supported by, at very least, the results of an imaging test. Disease response was assessed by positron emission tomography-computed tomography (PET-CT) in all but three patients. OS was defined as the time from alloSCT to death from any cause. PFS was defined as the time from alloSCT to the date of relapse, progression or death from any cause. The day of neutrophil and of platelet recovery was considered as the first of three consecutive days post-transplantation with an absolute neutrophil count >0.5 × 10^9^/L and a platelet count >20 × 10^9^/L, respectively. Acute (aGVHD) and chronic (cGVHD) GVHD were defined according to standard EBMT/NIH (Blood and Marrow Transplantation/National Institutes of Health) criteria [25,26]. NRM was defined as mortality secondary to any cause in the absence of prior relapse/progression of tFL. OS, PFS and NRM curves were estimated by the Kaplan–Meier method and compared using the log-rank test. For NRM, competitive risks were considered in the analysis. Multivariate Cox analyses were undertaken to investigate factors that might be predictors of survival. Analyses were performed using R version 4.0.3 (R Foundation for Statistical Computing, Vienna, Austria) and IBM SPSS Statistics version 25 (IBM Corporation, Armonk, NY, USA).

## 3. Results

### 3.1. Patient Characteristics

Patient characteristics are shown in Table 1. The median age was 44 (range, 22–64) years at the time of the FL diagnosis, 51 (30–62) years at the time of HT and 52 (31–66) years at the time of alloSCT. The median time from the diagnosis of FL to HT was 49 (0–379) months, and 17 (4–123) months from HT to alloSCT. All patients had been treated with immunochemotherapy prior to alloSCT. They had received a median of two (range, 0–5) lines of therapy for FL before HT and a median of two (1–4) lines after HT. Seven patients of the 25 for whom data were available were chemotherapy-naïve at the time of HT. Most of the patients (*n* = 33, 87%) had received at least three previous lines of treatment before alloSCT, including autoSCT in 24 of them (63%). None of the patients received a tandem transplant (autologous followed by allogeneic transplantation). We only have detailed information about the regimens employed in previous lines in 25 patients; the majority of them (*n* = 18, 72%) received R-CHOP (rituximab, cyclophosphamide, doxorubicin, vincristine, and prednisone) as the first-line. No patient received Rituximab-Bendamustine upfront, as it is not approved in Spain as a first-line treatment. However, six patients (24%) received this regimen as salvage therapy. Other commonly used regimens in the salvage setting are R-ESHAP (rituximab, etoposide, cytarabine, cisplatin and methylprednisolone, *n* = 18, 72%) and R-GEMOX (rituximab, gemcitabine, oxaliplatin and dexamethasone, *n* = 7, 28%). The median time from diagnosis to transplant was 46 (7–261) months for autoSCT and 61 (8–430) months for alloSCT. The hematopoietic cell transplantation-specific comorbidity index (HCT-CI) [27] was ≥3 in six patients (15.8%). Disease status at alloSCT was complete remission (CR) in 21 patients (55%), partial response (PR) in 12 patients (32%), and stable or progressive disease in four patients (11%). The status of one patient was not known.

Other characteristics, such as the status of the MYC gene have not been studied because in the first decade of the study it was not a standard practice.

With regard to the conditioning regimen, 28 patients (74%) received reduced-intensity conditioning (RIC) and 10 (26%) patients received myeloablative conditioning (MAC) (Table 1). Patients who received RIC conditioning had been previously treated with autoSCT or were >55 years old. FluMel was the most often used regimen. Eleven patients received anti-CD20 drugs (rituximab, *n* = 2; ibritumomab tiuxetan, *n* = 2; and ofatumumab, *n* = 7) as part of the conditioning regimen within clinical trials [28]. Other transplantation-related characteristics are summarized in Table 1.

### 3.2. Donors

The donor for 20 (53%) patients was an HLA-identical sibling, a matched-unrelated donor in eight (21%) patients, a mismatched relative in one (3%) patient, and a haploidentical donor in seven (18%) patients. Donor data were not available for two (5%) patients.

Thirty-six (95%) patients received hematopoietic stem cells collected from peripheral blood (PB). Stem cells were obtained from bone marrow (BM) and cord blood for one patient each (Table 1).

### 3.3. Engraftmet and Chimerism

Engraftment was achieved in all but one patient, with early mortality before day +30. Median times for neutrophil and platelet recovery were 15 (range, 9–156) days and 12 (6–369) days, respectively.

Chimerism data were available for 12 patients at day +21, +56 and +100 in BM and PB. At day +21, full donor chimerism was documented in nine (75%) patients in PB and in 10 (83%) patients in BM. All evaluable patients with available data had full donor chimerism at day +100 in both BM and PB.

### 3.4. Graft-Versus-Host Disease

Acute GVHD was observed in 26 (69%) patients, but only six (16%) patients developed grade III-IV disease. Thirty-two of thirty-eight patients were evaluable for cGVHD (alive at day +100), of whom twelve (38%) patients had developed cGVHD by a median of 216 (53–560) days post-alloSCT. It was mild in four patients, moderate in six, and severe in two patients.

### 3.5. Response and Relapse Rate

Post-transplant, twenty-six (68%) patients had a CR, and two (5%) patients had a PR, as shown in Table 2. The CR rate was 90% among patients with CR at alloSCT, 41% among those with PR, and 50% among those with SD/PD (*p* < 0.01). Interestingly, two patients who were in PR at alloSCT and maintained PR at day +100, achieved CR after lowering immunosuppression and donor lymphocyte infusion, respectively. In the overall series, nine (24%) patients relapsed or progressed after a median of 7 (3–56) months after alloSCT, and fourteen (37%) patients were alive and in CR after a median follow-up of 58 months.

### 3.6. Overall and Progression-Free Survival

**Figure 1:**
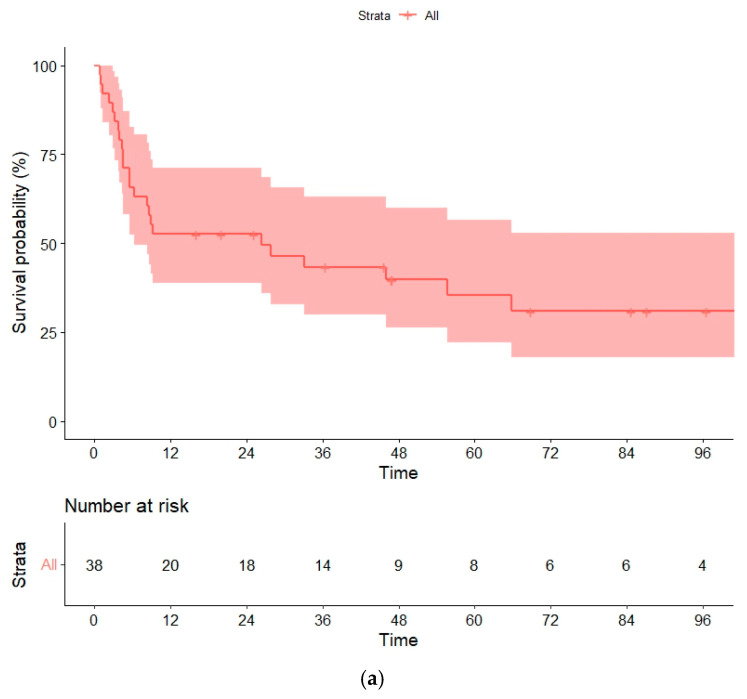

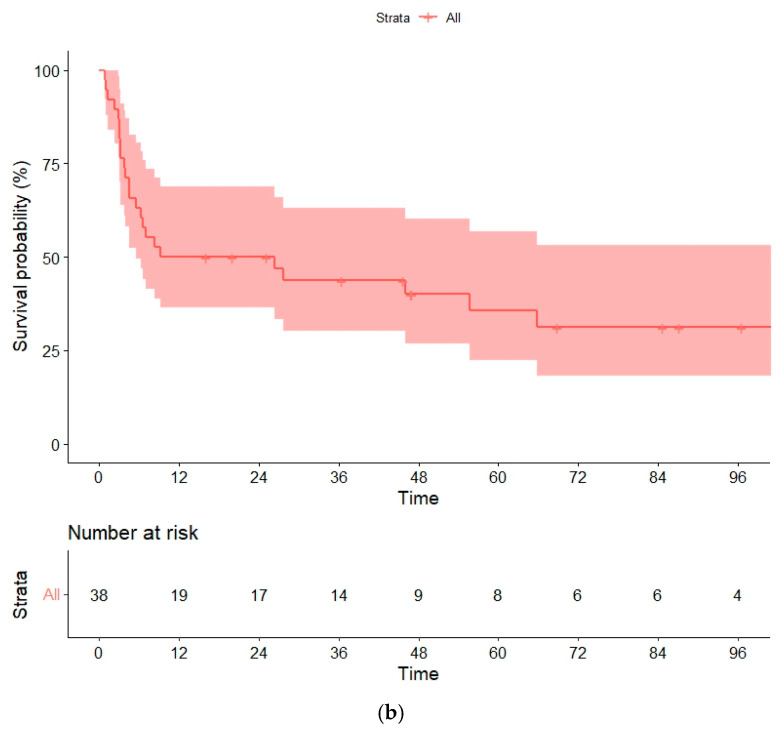
(**a**) Overall survival (OS) and (**b**) progression-free survival (PFS) in the global series.

With a median follow-up of 58 (16–134) months for survivor patients, the median OS was 26 (95% confidence interval [CI], 0–59) months, and the median PFS was 9 months (95% CI, 0–38). Estimated OSs and PFSs at 1, 3 and 5 years were 52% (95% CI, 43–60%), 43% (32–58%) and 35% (27–49%), and 50% (27–60.5%), 43% (19–51%), and 35% (19–52%), respectively (Figure 1a,b). The strong collinearity between relapse and death in our cohort meant that PFS and OS data were comparable.

**Figure 2:**
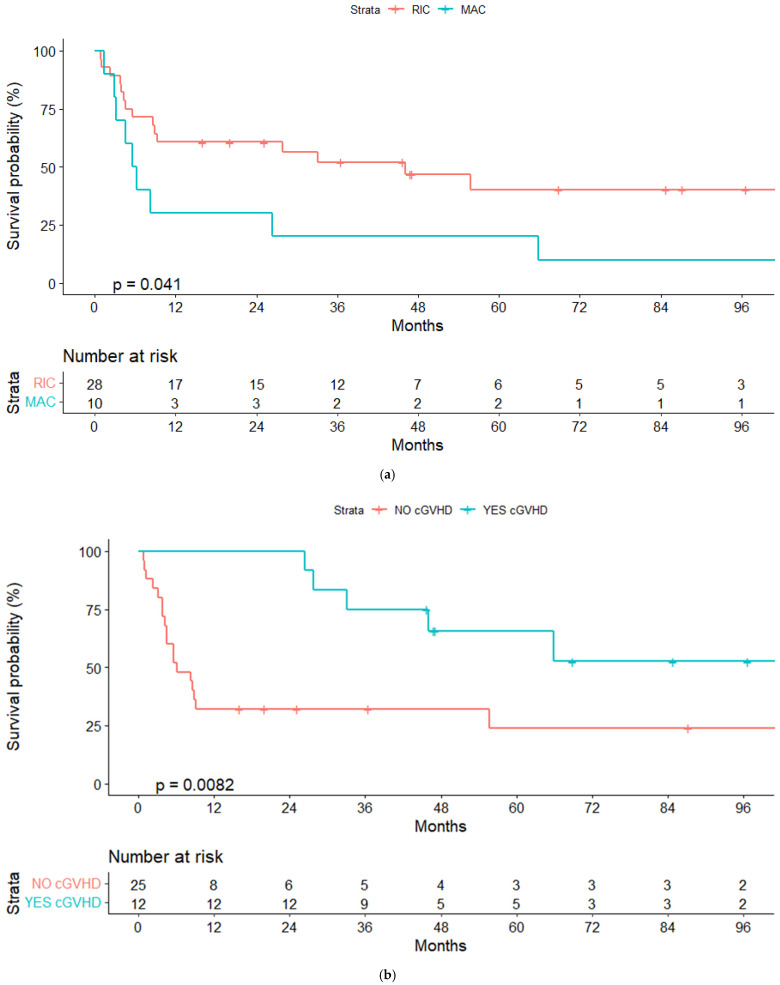

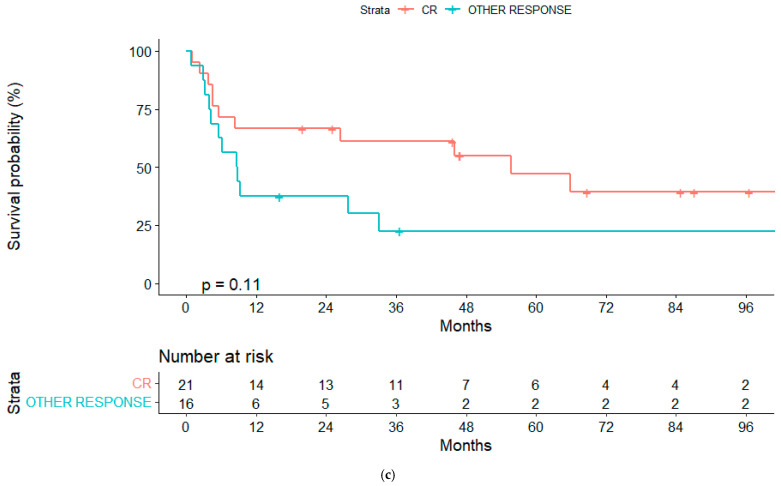
(**a**) Overall survival (OS) for reduced-intensity conditioning (RIC) and myeloablative conditioning (MAC) regimens. (**b**) OS according to chronic graft-vs-host disease (cGVHD). (**c**) OS according to lymphoma status pre-alloSCT, complete response (CR) or other response.

**Figure 3:**
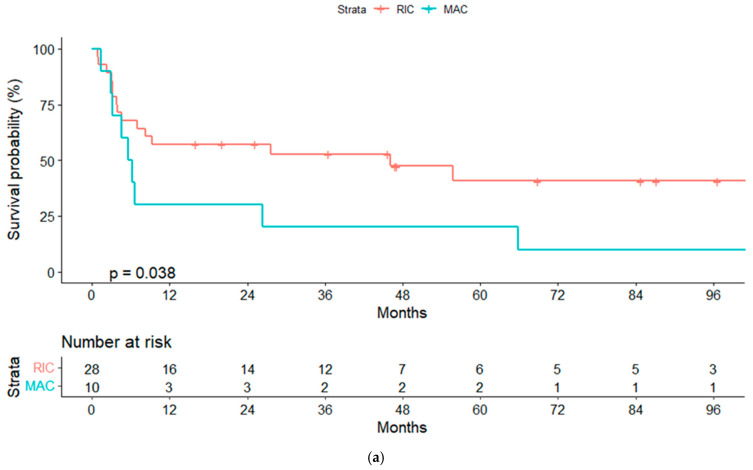

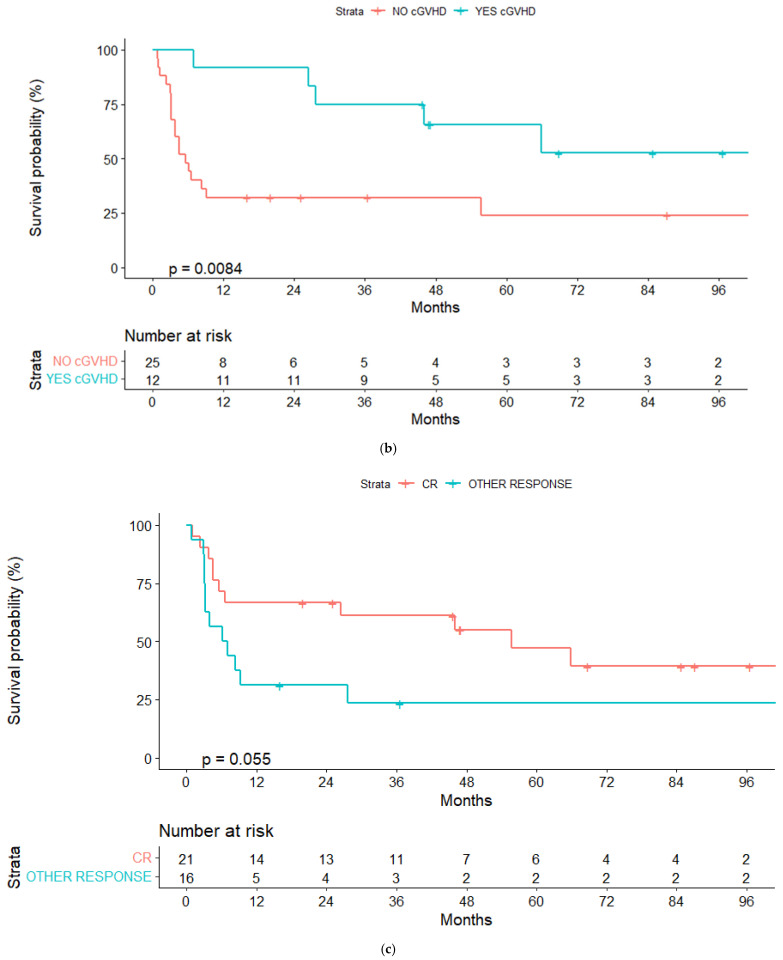
(**a**) Progression free-survival (PFS) for reduced-intensity conditioning (RIC) and myeloablative conditioning (MAC) regimens. (**b**) PFS according to chronic graft-vs-host disease (cGVHD). (**c**) PFS according to lymphoma status pre-alloSCT, complete response (CR) or other response.

In the univariate analyses, the conditioning regimen and development of cGVHD were the only factors significantly associated with OS (Table 3). Three-year OS estimates were 52% and 20%, respectively, for patients treated with the RIC and MAC regimen (*p* = 0.04), and 75% and 40%, respectively, for patients who developed or not cGVHD (landmark analysis of patients alive at day +100, *p* = 0.032) (Figure 2a,b and Figure 3a,b).

We also noted a trend towards better OS in patients who were treatment-naïve at the moment of HT (3-year OS, 71% vs. 49%, *p* = 0.15) and in those in CR pre-alloSCT (3-year OS, 61% vs. 23%, *p* = 0.1), suggesting that it would be worth examining these variables in a larger cohort.

Regarding the impact of the type of donor, we obtained similar results in patients receiving an alloSCT from an HLA-identical (related or unrelated) or from a haploidentical donor (3-year OS, 49.5 vs. 57.1%), although the three patients with a mismatched donor died (*p* = 0.14).

The only variable with an independent prognostic association with OS and PFS in a Cox regression model for patients alive at day +100 was the development of cGVHD (HR, 3.48; 95% CI, 1.22–9.96; *p* = 0.02; HR 2.97, 95% CI, 1.05–8.38; *p* = 0.04). When we considered only pre-alloSCT variables, none of them were significantly associated with OS or PFS.

### 3.7. Global Mortality and Non-Relapse Mortality

Twenty-four deaths were documented, nineteen of which occurred in the first 12 months post-transplant due to relapse or progression of the disease (*n* = 6) or NRM (*n* = 13). Disease progression was the main cause of death in the global series (n = 8, 33%), followed by infections (*n* = 7/10, 42%) and GVHD (*n* = 5/2, 21/5%). Other causes of death were sinusoidal obstructive syndrome (*n* = 1), syndrome myelodysplastic (*n* = 1), and secondary neoplasia (*n* = 1) (Table 2).

**Figure 4:**
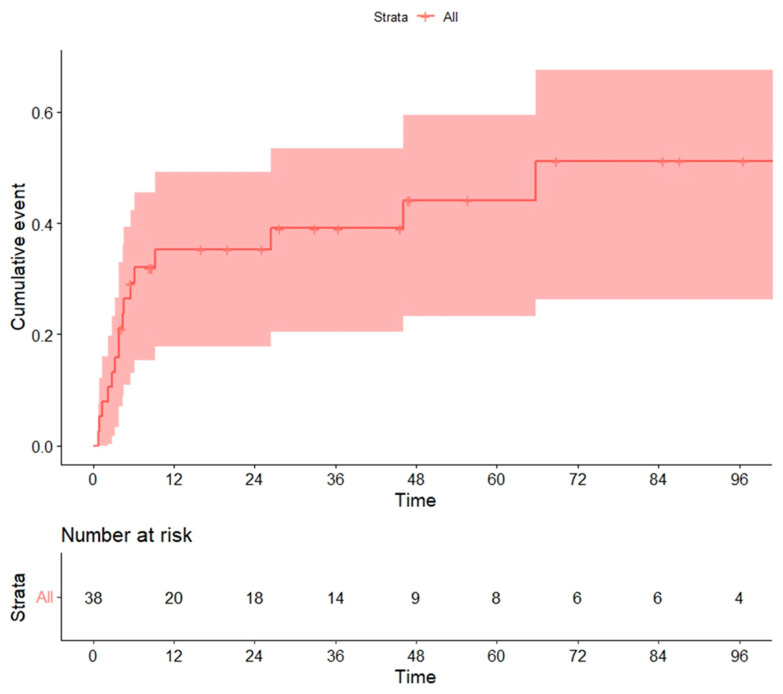
Non-relapse mortality (NRM) in global series.

**Figure 5:**
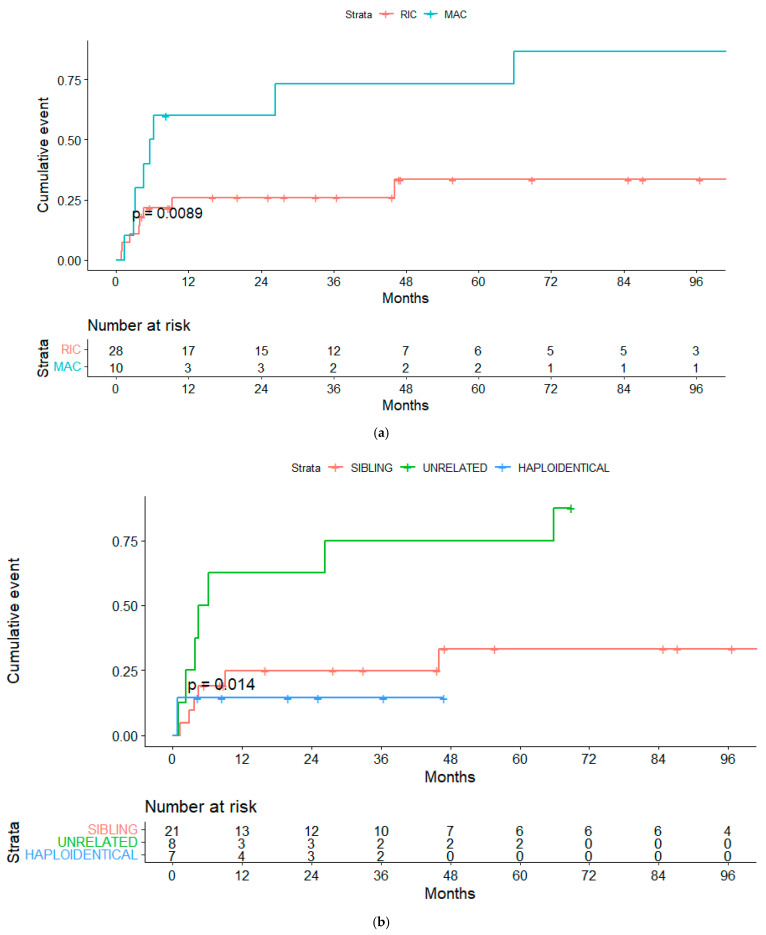
(**a**) Non-relapse mortality (NRM) for RIC and MAC regimens. (**b**) NRM for sibling vs. unrelated vs. haploidentical transplanta-tion.

Overall, 15 cases were considered NRM, resulting in a cumulative incidence of 20.5% at 100 days and 34% at 12 months (Figure 4). Variables significantly associated with global NRM were conditioning regimen and donor type. The NRM at 12 months (13/15 patients died during this period) was significantly higher in patients receiving the MAC rather than the RIC regimen (30% vs. 10.7%, *p* = 0.013) (Figure 5a), unrelated vs. related (including haploidentical) donors (62% vs. 26% vs. 14%, *p* = 0.018) (Figure 5b) or HLA-mismatched donor vs. HLA-identical (67% vs. 33%, *p* = 0.023).

Considering a Cox regression model for patients alive on day +100, the only variable with an independent prognostic association with NRM was the conditioning regimen (MAC vs. RIC) (HR, 3.63; 95% CI, 1.04–12.63; *p* = 0.04).

## 4. Discussion

Histological transformation represents a milestone in FL history, with a worse prognosis and fewer treatment options. AlloSCT continues to be one possible option, so specific analysis of alloSCT outcomes in patients with tFL is crucial to understanding their specific characteristics, especially those that may differ from those of de novo DLBCL or FL patients. However, few series focusing on these patients have been published; most of them were included within series of aggressive NHL, and no sub-analysis of tFL was undertaken.

Here we report a series of 38 patients with tFL receiving an alloSCT in Spain between 2000 and 2019. As far as we are aware, it is the largest series published to date that analyzes the outcome of this specific group of patients. Our primary aim was to evaluate the efficacy of alloSCT as a potential curative strategy for tFL, and we observed that, after a median follow-up of 60 months for living patients, the alloSCT made it possible for intensively pre-treated patients with limited therapeutic options to achieve long-term PFS and OS.

The results of other studies with fewer patients are similar to ours in terms of efficacy, although, unlike our study, other series included patients who had not been pre-treated with rituximab before their alloSCT. Heinzelmann et al. reported 2-year OS and PFS of 33% and 30%, respectively, in 33 patients with tFL who underwent alloSCT [24]. Rezvani et al. reported 3-year OS and PFS of 43% and 38%, respectively, in a smaller series (*n* = 16), which are comparable to our results [29]. As new findings, we confirmed the feasibility of haploidentical donor and post-alloSCT cyclophosphamide prophylaxis, specifically in those patients with tFL, with a similar outcome to those with HLA-identical donors (Table 4).

If we compare our data with those of other GELTAMO/GETH-TC group series in patients with FL or DLBCL who underwent alloSCT, we find that patients with FL present better survival rates, with 9-year OS and PFS of 58% and 53%, respectively [31]. However, those patients with de novo DLBCL have similar survival to patients with tFL (1-year and 3-year OS of 56% and 44%; 1-year and 3-year PFS of 49% and 38%). We conclude that patients with tFL have a similar post-alloSCT survival profile to patients with de novo DLBCL [32].

The greater curative potential of alloSCT relative to chemotherapy or autoSCT arises from the graft versus lymphoma effect [33]. Our study supports this effect, since development of cGVHD was associated with a significantly better OS, independent of other prognostic factors (HR 3.48, *p* = 0.02). Other series have shown that chemosensitive disease and the use of a RIC regimen were also associated with a favorable outcome [24]. Chemosensitive disease was not significantly associated with a better outcome in our cohort, suggesting that the graft-versus-lymphoma effect could lead to long-term response even in chemorefractory patients. However, this finding should be interpreted with caution due to the small numbers of patients available for the sub-analyses.

Unfortunately, alloSCT is associated with high morbidity and mortality, particularly in these heavily pre-treated patients. NRM in our series was 20.5% and 34.3% at 100 days and 1 year, respectively, significantly higher with MAC and unrelated donors. These data are comparable to those from other series with similar patient characteristics [24]. The NRM in our series seems higher than reported in FL patients without histological transformation, which is around 15% and 25% at day +100 and at 3 years after alloSCT, respectively [34,35], and seems similar to that reported in patients with DLBCL undergoing alloSCT [36].

In patients with DLBCL who have failed at least two lines, alloSCT and Chimeric Antigen Receptor (CAR) T-cells are both options. Current data indicate that CAR T-cells are preferable to transplantation because of their safety profile and documented efficacy in refractory disease [37]. However, the majority of patients who receive CAR T-cell therapy progress or relapse thereafter, so alloSCT could still be an option for these patients.

Our study had several strengths. Most importantly, it was based on a series of histologically confirmed tFLs treated in the rituximab era, and with a long median follow-up of almost 5 years. However, it also has some potential limitations. Although our series is the largest published in the field, the sample size is too small to identify statistically significant associations with prognostic factors that could help us select patients who would genuinely benefit from alloSCT. In addition, it has the limitations characteristic of a retrospective study of patients receiving alloSCT for 20 years. Different protocols for GVHD prophylaxis, donor selection and supportive care can generate study bias, and retrospective collection of data could underestimate cGVHD incidence, which was remarkably low in our series. In addition, biological variables of interest, such as MYC rearrangements at the time of transplant, were not available for most patients.

## 5. Conclusions

To conclude, our results indicate that alloSCT continues to be a potentially curative option for patients with tFL. In the current scenario, whereby various strategies such as CAR T-cells and new drugs are available, it is crucial to understand which factors are associated with a better outcome after each therapy, so that the best candidates for each treatment strategy can be selected. Our series provides data from a real-life study that could inform such a decision.

## Figures and Tables

**Table 1:** Patient and transplant-related characteristics (*n* = 38).

Characteristic		Frequency (Range or Percentage)
Age, median (range)		52 (31–66)
Sex, *n* (%)	Male	24 (63%)
	Female	14 (37%)
Previous lines of treatment, median (range)		4 (2–7)
Previous lines of treatment, *n* (%)	2	5 (13%)
	3	14 (37%)
	4 or more	19 (50%)
Previous ASCT, *n* (%)	Yes	24 (63%)
	No	14 (37%)
Lymphoma status pre-AlloSCT, *n* (%)	CR	21 (55%)
	PR	12 (32%)
	SD/PD	4 (11%)
	Data not available	1 (2%)
HCT-CI, *n* (%)	0	17 (44.7%)
	1	10 (26.3%)
	2	3 (7.9%)
	≥3	6 (15.8%)
	Data not available	2 (5.3)
HLA identical and donor, *n* (%)	Identical donor	26 (68%)
	Related	21 (56%)
	Unrelated	5 (12%)
	Haploidentical	7 (18%)
	Mismatched donor unrelated	3 (8%)
	Data not available	2 (5%)
Source of stem cells, *n* (%)	PB	36 (95%)
	BM	1 (2.5%)
	CBU	1 (2.5%)
Intensity conditioning regimen, *n* (%)	Myeloablative (MAC)	10 (23.3%)
	Reduced intensity (RIC)	26 (68.4%)
	Data not available	2 (5.3%)
Conditioning regimen, *n* (%)	Flu-Mel	21 (55.3%)
	Flu-Bu	4 (23.7%)
	Flu-Bu-Thio	4 (10.5%)
	CyTBI	1 (2.6%)
	Flu-TBI	2 (5.2%)
	Bu-Cy	1 (2.6%)
Monoclonal antibodies as part of conditioning regimen, *n* (%)	Rituximab	2 (5.3%)
	Ofatumumab	7 (18.4%)
	Ibritumomab tiuxetan	2 (5.3%)
GVHD prophylaxis, *n* (%)	MTX + CI	13 (34.2%)
	Tacro + Rapa	11 (28.9%)
	MMF + CI	4 (10.6%)
	Cy post-alloSCT	7 (18.4%)
	Alemtuzumab/ATG	2 (5.3%)

Abbreviations: ASCT, autologous stem cell transplantation; allo-SCT, allogeneic stem cell transplantation; CR, complete remission; PR, partial response; SD, stable disease; PD, progressive disease; HSC, hematopoietic stem cells; PB, peripheral blood; BM, bone marrow; CBU, cord blood unit; GVHD, graft-vs-host disease; Flu: fludarabine; Bu: busulfan; Thio: thiotepa; Cy; cyclophosphamide; TBI, total body irradiation; MTX, methotrexate; CI, calcineurin inhibitor: Tacro, tacrolimus, Rapa: rapamycin, MMF: Mofetil mycophenolate, ATG: Anti-thymocyte globulin.

**Table 2:** Response to transplantation and current status of patients.

Status at alloSCT	Response at Day +100	Status at Last Follow-Up	Cause of Death
CR, *n* = 21	CR, *n* = 19	Alive in CR, *n* = 10	
		Death while in CR, *n* = 8	Infection, *n* = 5 aGVHD *n* = 2 Secondary neoplasm, *n* = 1
		Death after progression, *n* = 1	Lymphoma, *n* = 1
	Relapse, *n* = 1	Death, *n* = 1	Lymphoma, *n* = 1
	Death without evaluation, *n* = 1	Death, *n* = 1	SOS, *n* = 1
PR, *n* = 12	CR, *n* = 5	Alive in CR, *n* = 1	
		Death in CR, *n* = 2	Infection, *n* = 2
		Death after progression, *n* = 2	Lymphoma, *n* = 2
	PR, *n* = 3	Alive in CR, *n* = 2 *	
		Death in CR, *n* = 1	NRM, *n* = 1
	SD/PD, *n* = 3	Death, *n* = 3	Lymphoma, *n* = 3
	Not evaluable, *n* = 1	Death after progression, *n* = 1	Lymphoma, *n* = 1
SD/PD, *n* = 4	CR, *n* = 2	Alive in CR, *n* = 1	
		Death in CR, *n* = 1	Infection, *n* = 1
	Death without evaluation, *n* = 2	Death, *n* = 2	Infection, *n* = 2
No data, *n* = 1	Death without evaluation, *n* = 1	Death, *n* = 1	MDS, *n* = 1

* One patient achieved CR after lowering immunosuppression and the other after donor lymphocyte infusions. Abbreviations: alloSCT: allogeneic stem cell transplantation; CR: complete remission; PR: partial response; SD: stable disease; PD: progression disease; aGVHD: acute graft-vs-host disease, SOS: sinusoidal obstructive syndrome; NRM: non-relapse mortality; MDS: myelodysplastic syndrome.

**Table 3:** Univariate analysis of survival.

Univariate Analysis				
Characteristics	3y-PFS (95% CI)	*p*	3y-OS (95% CI)	*p*
Age at alloSCT:				
<59	45% (28–62)		44.6% (27.4–61.8)	
>60	30% (16.9–76.9)	0.86	30% (16.8–76.8)	0.75
Previous lines of therapy:				
1–3	39.5% (16.4–62.6)		39.5% (16.4–62.7)	
>3	47.4% (24.9–69.9)	0.95	46.80%	0.95
Previous ASCT:				
Yes	56.4% (35.8–77)		56.4% (36–77)	
No	21.4% (0.2–43)	0.06	21.4% (0.1–43)	0.13
Conditioning regimen:				
MAC	20% (4.7–44.7)		20% (5–45)	
RIC	52.7% (33.9–71.5)	0.04	52% (33–71)	0.04
Donor:				
Sibling	52% (30.3–73.5)		51.6% (29.8–73.4)	
Haploidentical	57% (20.4–93.8)		57% (20.4–93.8)	
Unrelated	25% (5–55)	0.3	25% (5–55)	0.22
Response pre alloSCT:				
CR	61% (39.9–82.3)		66.7% (46.5–86.9)	
Other	23.4% (1.8–45)	0.06	22.5% (1–44)	0.11
aGVHD:				
Yes	41.5% (22.3–60.7)		41% (21.5–60.3)	
No	53% (22.6–83.4)	0.66	53% (22.6–83.4)	0.67
aGVHD:				
0–2	47% (46.8–47.2)		46.5% (27.4–63.8)	
>2	33.3% (31.9–33.7)	0.9	33.3% (4.3–70.9)	0.65
cGVHD:				
Yes	75% (50.5–99.5)		75% (50.5–99.5)	
No	32% (13.8–50.2)	0.008	32% (13.8–50.2)	0.008

Abbreviations: PFS: progression-free survival; OS: overall survival; ASCT, autologous stem cell transplantation; MAC: myeloablative conditioning; RIC: reduced-intensity conditioning; allo-SCT, allogeneic stem cell transplantation; CR, complete remission; aGVHD: acute GVHD; cGVHD: chronic GVHD.

**Table 4:** Previously published series.

Reference	Patients (n)	CR (%)	OS/PFS (%)	NRM (%)
Heinzelman et al., J. Cancer Res. Clin. Oncol. 2018 [24]	33	62%	1-y: 49/33 2-y: 39/30 5-y: 33/24	30-d: 9 100-d: 25 1-y: 38 2-y: 43
Rezvani et al., Clin. Oncol. 2008 [29]	16	-	3-y OS: 43 3-y PFS: 38	3-y: 42
Villa et al., Clin. Oncol. 2013 [13]	22	59%	5-y OS: 45	5-y: 23
Wirk et al., BBMT 2014 PMID: 24641828 [30]	33	-	5-y: 22/18	1-y: 41

## Data Availability

The data presented in this study are available on request from the corresponding author.
